# T-bet, but not Gata3, overexpression is detrimental in a neurotropic viral infection

**DOI:** 10.1038/s41598-017-10980-0

**Published:** 2017-09-05

**Authors:** Fumitaka Sato, Eiichiro Kawai, Nicholas E. Martinez, Seiichi Omura, Ah-Mee Park, Satoru Takahashi, Keigyou Yoh, Ikuo Tsunoda

**Affiliations:** 1 Department of Microbiology, Kindai University Faculty of Medicine, 377-2 Ohnohigashi, Osakasayama, Osaka 589-8511 Japan; 2 Department of Microbiology and Immunology, Louisiana State University Health Sciences Center-Shreveport (LSUHSC-S), 1501 Kings Highway, Shreveport, LA 71130 USA; 3 Center for Molecular and Tumor Virology (CMTV), Louisiana State University Health Sciences Center-Shreveport (LSUHSC-S), 1501 Kings Highway, Shreveport, LA 71130 USA; 4 Center for Cardiovascular Diseases and Sciences (CCDS), Louisiana State University Health Sciences Center-Shreveport (LSUHSC-S), 1501 Kings Highway, Shreveport, LA 71130 USA; 5 Department of Anatomy and Embryology, Faculty of Medicine, University of Tsukuba, 1-1-1 Tennodai, Tsukuba, Ibaraki 305-8575 Japan; 6 International Institute for Investigative Sleep Medicine (WPI-IIIS), University of Tsukuba, 1-1-1 Tennodai, Tsukuba, Ibaraki 305-8575 Japan; 7 Life Science Center, Tsukuba Research Alliance (TARA), University of Tsukuba, 1-1-1 Tennodai, Tsukuba, Ibaraki 305-8575 Japan; 8 Laboratory Animal Resource Center (LARC), University of Tsukuba, 1-1-1 Tennodai, Tsukuba, Ibaraki 305-8575 Japan

## Abstract

Intracerebral Theiler’s murine encephalomyelitis virus (TMEV) infection in mice induces inflammatory demyelination in the central nervous system. Although C57BL/6 mice normally resistant to TMEV infection with viral clearance, we have previously demonstrated that RORγt-transgenic (tg) C57BL/6 mice, which have Th17-biased responses due to RORγt overexpression in T cells, became susceptible to TMEV infection with viral persistence. Here, using T-bet-tg C57BL/6 mice and Gata3-tg C57BL/6 mice, we demonstrated that overexpression of T-bet, but not Gata3, in T cells was detrimental in TMEV infection. Unexpectedly, T-bet-tg mice died 2 to 3 weeks after infection due to failure of viral clearance. Here, TMEV infection induced splenic T cell depletion, which was associated with lower anti-viral antibody and T cell responses. In contrast, Gata3-tg mice remained resistant, while Gata3-tg mice had lower IFN-γ and higher IL-4 production with increased anti-viral IgG1 responses. Thus, our data identify how overexpression of T-bet and Gata3 in T cells alters anti-viral immunity and confers susceptibility to TMEV infection.

## Introduction

Based on the differences of cytokine profiles, CD4^+^ T cells are classified into four subsets: T helper (Th) 1, Th2, Th17, and regulatory T cells (Tregs)^1, 2^. The following transcription factors contribute to the differentiation of murine Th cell subsets^3^: T-box transcription factor TBX21 (T-bet) for Th1 cells^4, 5^, GATA binding protein 3 (Gata3) for Th2 cells^6, 7^, retinoic-acid-receptor-related orphan receptor-γt (RORγt) for Th17 cells, and forkhead box P3 (Foxp3) for Tregs^8–10^. Among the Th cell subsets, Th1 and Th2 cells play protective roles in viral infections^11–13^. Th1 cells help cellular anti-viral immunity by producing interferon (IFN)-γ and interleukin (IL)-2, while Th2 cells help humoral anti-viral immunity by producing IL-4, IL-5, and IL-13^14, 15^. In some cases, however, Th1 and Th2 cells can play pathogenic roles in viral infections^16^. While uncontrolled Th1 cells can cause immune-mediated tissue damage (immunopathology)^17^, increased Th2 cells can also induce tissue damage by increasing viral replication and/or persistence (viral pathology), since Th2 cells suppress Th1-mediated anti-viral cellular immunity^18^. These findings have been mainly based on “loss-of-function” experiments, using either blocking antibodies against Th1/Th2 cytokines or knockout (KO) mice lacking Th1/Th2-related molecules.

A line of clinical reports have shown that “gain-of-function” mutations in signal transducer and activator of transcription 1 (*STAT*1) affect Th cell functions and alter susceptibility to autoimmune diseases and infections with microbes, including viruses^19–22^. Thus, we have established novel T-bet-transgenic (tg) mice and Gata3-tg mice that overexpress T-bet and Gata3 in T cells, as “gain-of-function” models^23^. Using these tg mice, we have demonstrated that Th1/Th2-related “gain-of-function” mutations alter susceptibility to some disease models^24, 25^. In naïve T-bet-tg mice, T-bet overexpression favors generation of IFN-γ-producing CD4^+^ Th1 and CD8^+^ T cells, while the effects on immune-mediated diseases differ depending on disease models. T-bet-tg mice have disease exacerbation in animal models for contact dermatitis and glomerulonephritis, compared with wild-type mice^26, 27^. In contrast, in a collagen-induced arthritis (CIA) model, T-bet-tg mice do not have increased IFN-γ expression, while T-bet-tg mice neither develop CIA nor produce anti-collagen antibody, the latter of which is associated with suppression of Th17 cells^28^. On the other hand, Gata3-tg mice become susceptible to Th2-mediated diseases, such as allergic airway inflammation and pulmonary fibrosis, compared with wild-type mice^29, 30^. In viral infections, however, the influence of Th1 or Th2-biased responses remains unclear.

Theiler’s murine encephalomyelitis virus (TMEV) is a non-enveloped, positive-sense, single-stranded RNA virus that belongs to the family *Picornaviridae* ^31, 32^. TMEV is divided into two subgroups, George’s disease 7 (GDVII) and Theiler’s original (TO), based on the neurovirulence^33^. After intracerebral injection, the neurovirulent GDVII subgroup viruses cause fatal acute polioencephalomyelitis regardless of mouse strains; most GDVII virus-infected mice die by 10 days post infection (p.i.). Since GDVII virus-infected mice cannot mount anti-viral immune responses, GDVII virus infection is considered to be a pure viral pathology model^34, 35^.

Intracerebral injection of less virulent TO subgroup viruses, including the Daniels (DA) and BeAn strains, does not cause fatal infection in any immunocompetent wild-type mouse strains^31, 36^. During the acute phase, all the mouse strains induce anti-viral immune responses and develop mild neurological signs^32, 34^. During the chronic phase, SJL/J mice that are a susceptible mouse strain develop TMEV-induced demyelinating disease (TMEV-IDD) in the central nervous system (CNS), which is mainly caused by immunopathology. Since TMEV-IDD resembles multiple sclerosis (MS) in humans clinically and histologically^37, 38^, DA or BeAn virus infection in SJL/J mice has been widely used as a viral model of MS. In contrast, resistant mouse strains, such as C57BL/6 mice, have no inflammation with complete viral clearance in 2–3 weeks p.i.^39–41^. The different susceptibility to TMEV-IDD has been proposed to result from the different Th responses among mouse strains^42, 43^.

Resistant mouse strains, particularly C57BL/6 mice, have been used to clarify the key factors that are responsible for viral clearance as well as immunopathology by blocking and modulating key molecules and immune cells to see whether such immunomodulation could alter susceptibility to TMEV infection. For example, major histocompatibility complex (MHC) class I-deficient mice on the resistant mouse background have been shown to become susceptible to TMEV-IDD, suggesting that CD8^+^ T cells play a key role to eradicate TMEV^44–47^. Using RORγt-tg mice that overexpress RORγt in T cells, we previously demonstrated that an increase in Th17 responses rendered C57BL/6 mice susceptible to TMEV-IDD^48^. TMEV-infected RORγt-tg mice had viral persistence, higher levels of IL-17 production, lower levels of IFN-γ production, and fewer CD8^+^ T cells without alteration in overall levels of anti-TMEV lymphoproliferative and antibody responses during the chronic phase. On the other hand, RORγt-tg mice developed a CNS disease similar to wild-type mice during the acute phase of TMEV infection clinically and histologically. Intracerebral injection of TMEV has also been used as a viral model of seizures/epilepsy, since C57BL/6 mice, but not SJL/J mice, develop seizures during the first week of infection^41, 49^. In TMEV-induced seizures, the precise pathomechanism remains unclear.

The roles of Th1/Th2 immune responses in TMEV infection have been mainly investigated in mice whose Th1/Th2 cells were suppressed by “loss-of-function” approaches. These raised questions as to whether an increase in Th1/Th2 responses affects TMEV infection. We hypothesized that enhancement of Th1/Th2 responses could result in either a beneficial or detrimental outcome in TMEV infection by modulating anti-viral immune responses. In the present study, to determine the roles for T-bet and Gata3 overexpression in TMEV infection, we inoculated wild-type mice, T-bet-tg mice, and Gata3-tg mice on the resistant C57BL/6 mouse background with TMEV. Following intracerebral injection of less virulent DA virus, wild-type mice survived and did not develop TMEV-IDD. In contrast, T-bet-tg mice died of acute viral infection with lower levels of anti-viral humoral and cellular immune responses, which was associated with splenic T cell depletion. Gata3-tg mice remained resistant with a Th2-biased cytokine profile and increased anti-viral IgG1 production. Thus, T-bet overexpression may be detrimental in neurotropic viral infections.

## Results

### T-bet-tg mice die of infection with a less virulent strain of TMEV

**Figure 1:**
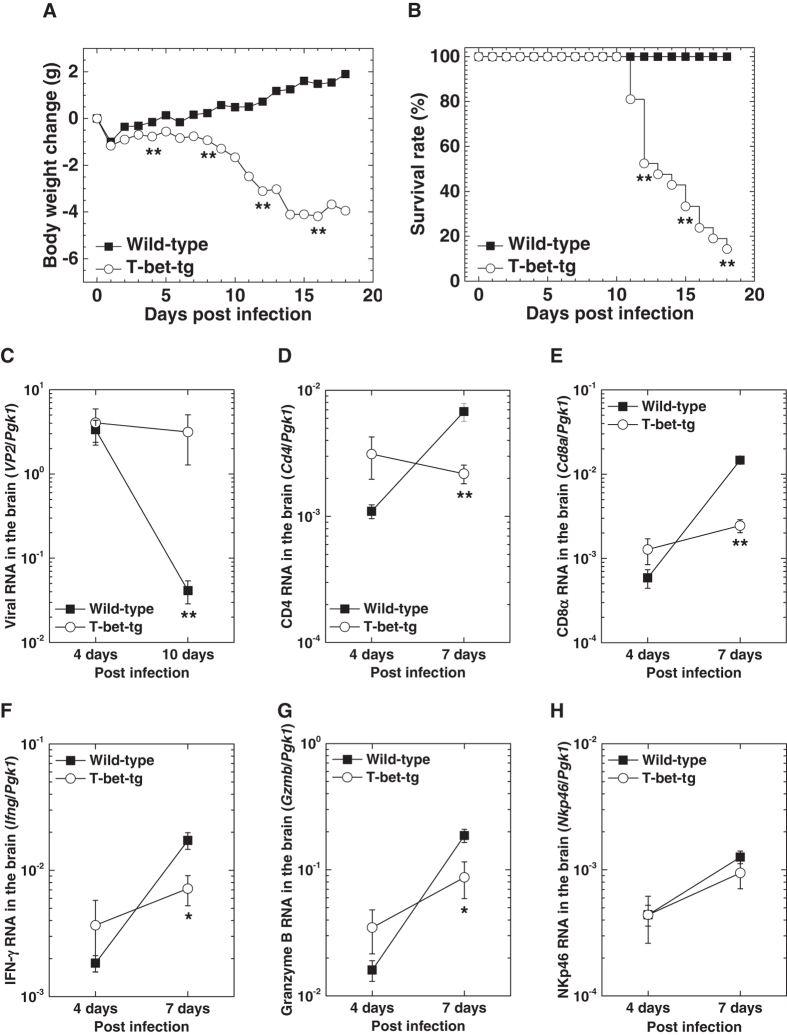
T-bet overexpression was detrimental in Theiler’s murine encephalomyelitis virus (TMEV) infection. (**A**) Body weight changes of wild-type mice (closed boxes) and T-bet-transgenic (tg) mice (open circles) after infection with the Daniels (DA) strain of TMEV (DA virus). **P < 0.01, Student *t* test. (**B**) Survival rates of wild-type mice and T-bet-tg mice infected with DA virus. **P < 0.01, chi-square (χ^2^) test. (**C**) Viral loads in the brains from wild-type mice and T-bet-tg mice 4 and 10 days after DA virus infection. **P < 0.01, Student *t* test. (**D**–**H**) Real-time polymerase chain reaction (PCR) analyses of *Cd4* (CD4^+^ T cell marker), *Cd8a* (CD8^+^ T cell marker), *Ifng* [interferon (IFN)-γ], *Gzmb* (granzyme B), and *Nkp46* (NK cell marker) in the brains from wild-type mice and T-bet-tg mice 4 and 7 days after DA virus infection. *P < 0.05 and **P < 0.01, Student *t* test. (**A**, **B**) Values are the mean of 20 wild-type mice and 21 T-bet-tg mice from three independent experiments. (**C**–**H**) Values are the mean ± standard error of the mean (SEM) of four to eight mice per time point.

To determine whether T-bet overexpression could alter susceptibility to TMEV infection, we infected wild-type mice and T-bet-tg mice on the C57BL/6 mouse background with the DA strain of TMEV (DA virus). Since DA virus does not cause fatal infection regardless of mouse strains^40, 48^, wild-type mice lost weight within a few days after DA virus infection and then gained weight with a 100% survival rate (Fig. 1A,B). In contrast, following DA virus infection, T-bet-tg mice had significant weight loss compared with wild-type mice 1 week p.i. and began to die 11 days p.i. After DA virus infection, 13 of the 15 (87%) T-bet-tg mice died with severe neurological signs; most T-bet-tg mice had hunched back and hind limb paresis followed by paraplegia, while some mice also developed spastic paralysis and priapism.

Since TMEV induces seizures in C57BL/6 mice during the first week of infection^49^, we monitored the clinical signs of seizures in DA virus-infected mice. The incidence and maximum scores were similar between DA virus-infected wild-type mice and T-bet-tg mice [incidence: wild-type, 73% (29 of 40 mice); T-bet-tg, 62% (23 of 37 mice), *P* = 0.3, chi-square (χ^2^) test; mean maximum Racine scale^50, 51^ scores ± standard error of the mean (SEM) in seized mice: wild-type, 5.0 ± 0; T-bet-tg, 4.7 ± 0.1].

### T-bet-tg mice have higher viral replication with lower anti-TMEV immune responses

To address the cause of death in T-bet-tg mice after DA virus infection, we semi-quantified viral genome in the brains of wild-type mice and T-bet-tg mice 4 and 10 days p.i. using real-time polymerase chain reaction (PCR) for a pair of primers against the capsid protein *VP2* of TMEV. Both wild-type mice and T-bet-tg mice had comparable viral replication in the brain 4 days p.i. (Fig. 1C). However, 10 days p.i., although wild-type mice had a significant decrease in viral replication, T-bet-tg mice had similar levels of viral RNA in the brain between 4 and 10 days p.i. In anti-viral cellular immunity, natural killer (NK) cells play a key role early, while T cell responses are observed as early as 5 days p.i. Thus, our results suggest that alteration of anti-viral T cells, but not NK cells, was likely responsible for the failure of viral clearance 10 days p.i. in T-bet-tg mice. While T cell-specific T-bet overexpression in T-bet-tg mice would mainly affect the generation of CD4^+^ Th1 and CD8^+^ T cells^4, 26, 52^, we quantified RNA expression of *CD4* (CD4^+^ T cell marker), *Cd8a* (CD8^+^ T cell marker), *Ifng* (interferon-γ), *Gzmb* (granzyme B), as well as *Nkp46* (NK cell marker) in the brain 4 and 7 days p.i. We found no significant differences in any of these RNA levels between wild-type mice and T-bet-tg mice, 4 days p.i. when NK cells play a central role in viral clearance (Fig. 1D–H); this is consistent with similar viral RNA levels between wild-type mice and T-bet-tg mice 4 days p.i. On the other hand, 7 days p.i. when T cells play a key role in viral clearance, T-bet-tg mice had significantly lower expression of *CD4*, *Cd8a*, *Ifng*, and *Gzmb*, but not *Nkp46*, compared with wild-type mice.

**Figure 2:**
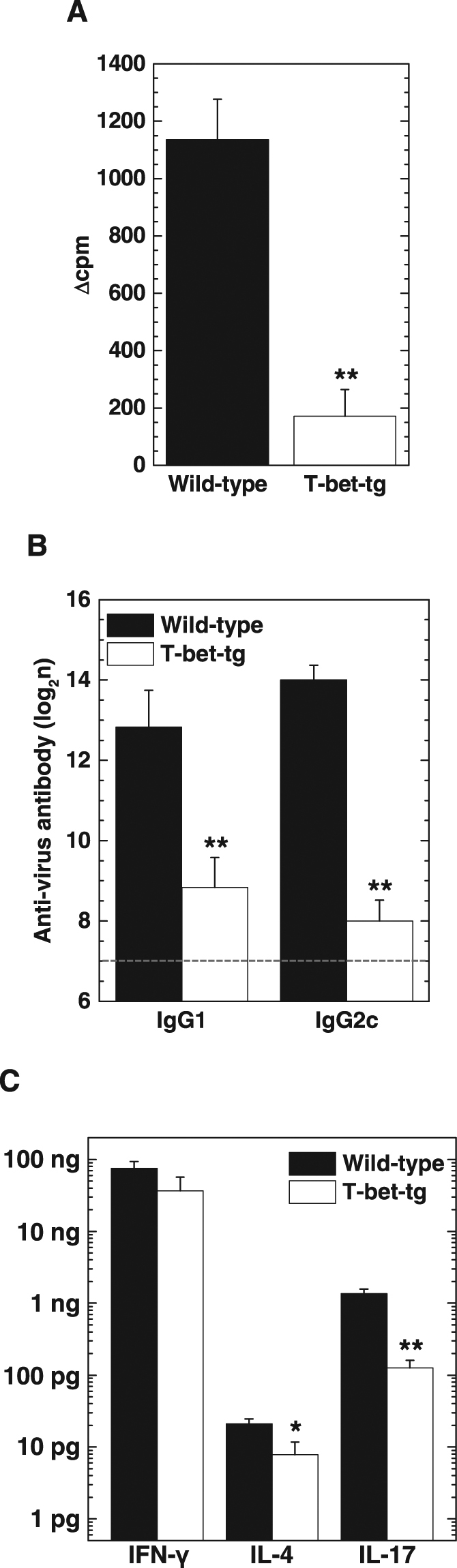
T-bet overexpression decreased anti-viral immune responses and production of interleukin (IL)-4 and IL-17. (**A**) TMEV-specific lymphoproliferative responses of splenic mononuclear cells (MNCs) from wild-type mice (black bar) and T-bet-tg mice (white bar) 10 days after DA virus infection. Values of lymphoproliferative responses to TMEV are expressed as Δcpm: (mean of experimental cpm in TMEV-specific stimulation) − (mean of control cpm). Values are the mean + SEM of three pools of spleens from two mice (six mice per group). (**B**) Enzyme-linked immunosorbent assays (ELISAs) of anti-TMEV IgG1 and IgG2c subclasses in sera from wild-type mice and T-bet-tg mice10 days after DA virus infection. The dotted line shows the detection limit. Values are the mean + SEM of six mice per group. (**C**) ELISAs of IFN-γ, IL-4, and IL-17 production from mitogen-stimulated splenic MNCs of DA virus-infected wild-type mice and T-bet-tg mice at day 10. Values are the mean + SEM from two independent experiments (five to six mice per group per experiment). (**A**–**C**) The experiments were conducted twice independently. *P < 0.05 and **P < 0.01, Student *t* test.

We quantified TMEV-specific lymphoproliferative responses (cellular immunity) and anti-TMEV antibody titers (humoral immunity) in wild-type mice and T-bet-tg mice 10 days p.i. using [^3^H]thymidine incorporation assays and enzyme-linked immunosorbent assays (ELISAs), respectively. The levels of TMEV-specific lymphoproliferation were significantly lower in T-bet-tg mice than in wild-type mice (Fig. 2A). T-bet-tg mice also had significantly lower titers of anti-TMEV IgG1 and IgG2c subclasses, compared with wild-type mice (Fig. 2B).

We also quantified the amounts of IFN-γ (Th1 cytokine), IL-4 (Th2 cytokine), and IL-17 (Th17 cytokine) production from splenic mononuclear cells (MNCs) of wild-type mice and T-bet-tg mice 10 days p.i. using ELISAs. Splenic MNCs from both wild-type mice and T-bet-tg mice had substantial production of IFN-γ (Fig. 2C). In contrast, the amounts of IL-4 and IL-17 production from splenic MNCs were statistically lower in T-bet-tg mice, compared with wild-type mice.

### T-bet-tg mice develop atrophy of the splenic T cell zone

**Figure 3:**
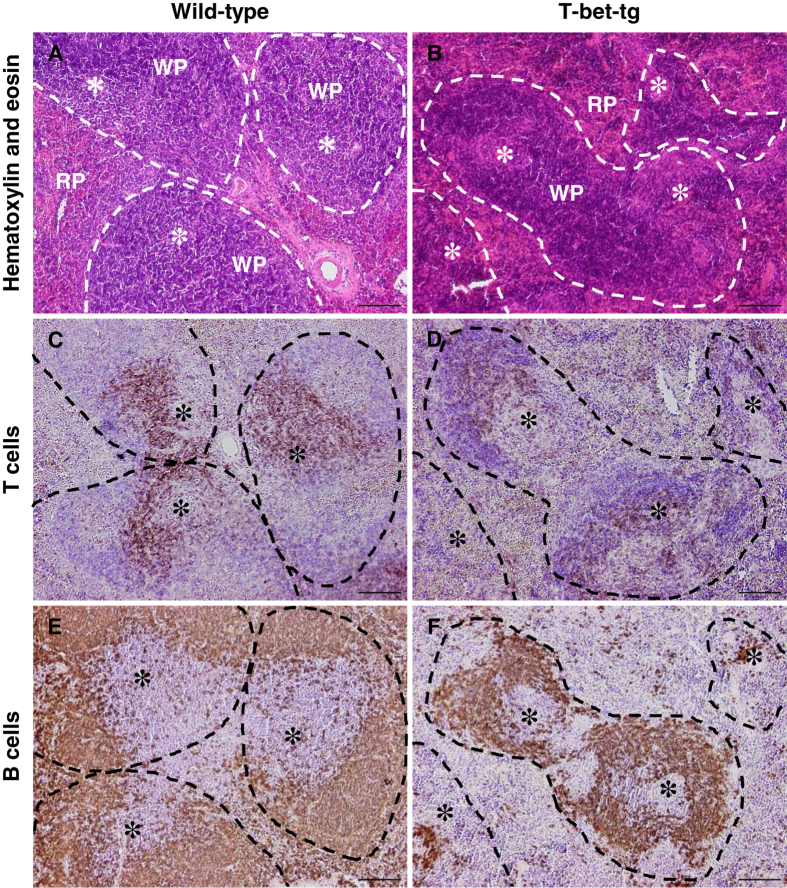
DA virus infection induced the change in spleen immune-architecture in T-bet-tg mice, but not in wild-type mice. (**A**,**B**) Hematoxylin and eosin stain showed that DA virus-infected T-bet-tg mice had small follicles (white pulp, WP, dotted line) with depletion of cells in the periarterial lymphoid sheath (PALS) (**B**), which formed a mass around the central artery (*) in DA virus-infected wild-type mice (**A**). (**C–F**) Immunohistochemistry against CD3 (T cell marker) and immunohistochemistry against B220 (B cell marker) showed depletion of T cells in the PALS with relative preservation of B cells in the white pulp in DA virus-infected T-bet-tg mice (**D**,**F**), compared with DA virus-infected wild-type mice (**C**,**E**). Spleen tissue sections (scale bar = 100 μm) were from DA virus-infected wild-type mice and T-bet-tg mice. RP, red pulp. Each group was composed of six to ten mice. Tissue sections are representative of two independent experiments.

Since we found impaired anti-TMEV cellular responses in the spleen with lower titers of anti-TMEV antibodies in DA virus-infected T-bet-tg mice, we conducted histological examinations of the spleen in DA virus-infected wild-type mice and T-bet-tg mice using hematoxylin and eosin staining. DA virus-infected wild-type mice had normal morphology of the splenic red pulp and white pulp, the latter of which was composed of the B cell zone as well as the T cell zone in the periarterial lymphoid sheath (PALS) surrounding the central artery (Fig. 3A). On the other hand, following DA virus infection, T-bet-tg mice developed substantial atrophy of the splenic white pulp (Fig. 3B). Immunohistochemical staining for CD3 (T cell marker) and B220 (B cell marker) showed that the white pulp atrophy of T-bet-tg mice was due to substantial depletion of CD3^+^ T cells in the PALS with relative preservation of B cells, compared with wild-type mice (Fig. 3C–F). There was no substantial difference in F4/80^+^ macrophages in the red pulp between wild-type mice and T-bet-tg mice after DA virus infection (Supplementary Fig. 1). Since no histological changes were seen in the spleens of uninfected T-bet-tg mice (Supplementary Fig. 2), the depletion of T cells was likely triggered by DA virus infection.

**Figure 4:**
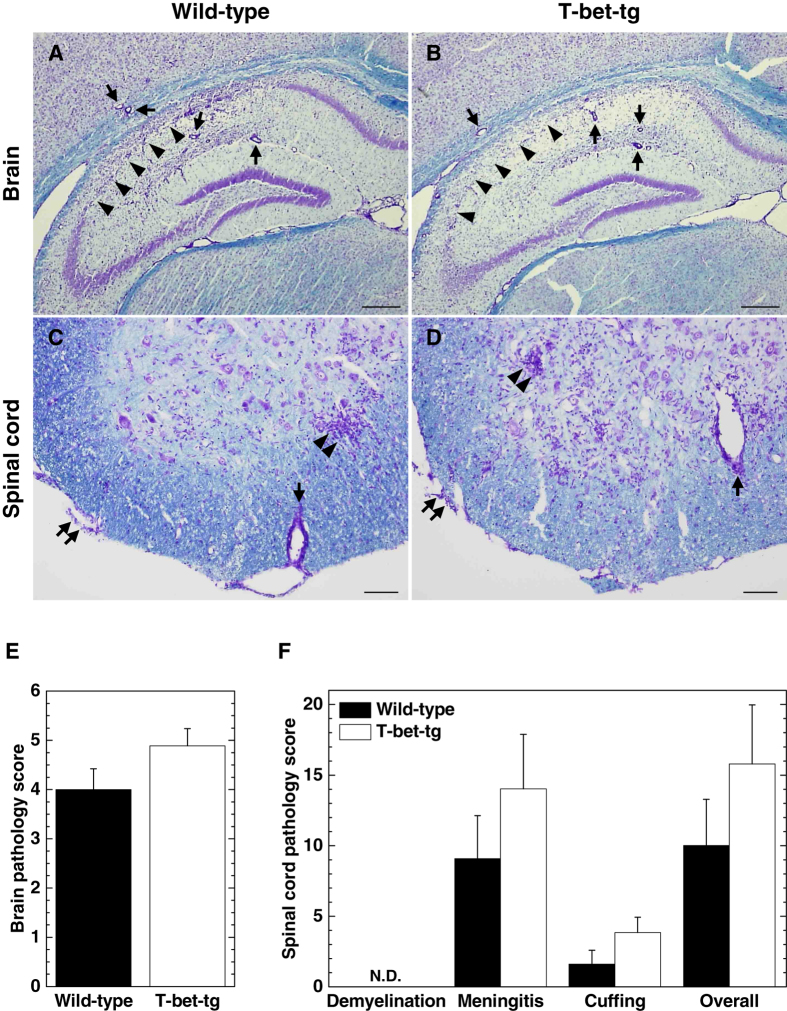
T-bet overexpression did not alter neuropathology in TMEV infection. (**A**–**D**) Luxol fast blue stains of the central nervous system (CNS) tissue sections from wild-type mice and T-bet-tg mice 10 days after DA virus infection. In the brain sections (scale bar = 300 μm), arrows and arrowheads show perivascular cuffing and neuronal loss, respectively. In the spinal cord sections (scale bar = 200 μm), paired arrows, arrows, and paired arrowheads show meningitis, perivascular cuffing, and neuronophagia, respectively. Tissue sections are representative of two independent experiments. (**E**,**F**) Pathology scores of the CNS tissue sections from DA virus-infected wild-type mice (black bar) and T-bet-tg mice (white bar) at day 10. N.D., not detectable. The experiments were conducted twice independently. Values are the mean + SEM of eight wild-type mice and nine T-bet-tg mice.

Since T-bet-tg mice began to die 11 days p.i. (Fig. 1B), we harvested the CNS tissues of wild-type mice and T-bet-tg mice 10 days p.i. and compared the neuropathology between the two groups. Four-μm-thick sections of the brain and spinal cord were stained with Luxol fast blue to visualize the myelin and inflammation. In the brain, particularly in the hippocampus, both wild-type mice and T-bet-tg mice had severe neuronal loss with mild inflammation (Fig. 4A,B). The brain inflammation scores 10 days p.i. were similar between wild-type mice and T-bet-tg mice (Fig. 4E). In the spinal cords of the two groups, we found mild meningitis, perivascular cuffing (inflammation), and microglial nodules, but not demyelinating lesions (Fig. 4C,D). There were no statistical differences in the spinal cord pathology scores between the two groups (Fig. 4F).

### T-bet overexpression does not alter susceptibility to a neurovirulent strain of TMEV

Since we did not find increased CNS inflammation in T-bet-tg mice in the above experiments, immunopathology would not be the cause of death in T-bet-tg mice following DA virus infection. To see viral pathology alone could result in the fatality of T-bet-tg mice after TMEV infection, we infected wild-type mice and T-bet-tg mice with 0.1, 1, 10, or 100 plaque forming units (PFUs) of the GDVII strain of TMEV (GDVII virus), which is neurovirulent. Following GDVII virus infection, mice cannot mount anti-viral acquired immune responses; infected mice die of viral pathology. In GDVII virus infection with 1 to 100 PFUs, most mice had encephalitic signs, such as weight loss, ruffled fur, and a hunched posture, around 6 days p.i. and died within 10 days p.i. (Table 1). The fatality, survival periods, and lethal dose (LD)_50_ titers were similar between wild-type mice and T-bet-tg mice (LD_50_ titers: wild-type, 0.40 PFUs; T-bet-tg, 0.24 PFUs). These results support that T-bet-tg mice would succumb to viral pathology, but not immunopathology, after DA virus infection as well as GDVII virus infection.

**Table 1:** Mortality and survival days of wild-type mice and T-bet-tg mice in GDVII virus infection.

PFU^b^	Wild-type^a^		T-bet-tg	
	Mortality^c^	Survival days ± SEM^d^	Mortality	Survival days ± SEM
100	4/4	6.0 ± 0.4	4/4	6.5 ± 0.3
10	11/11	6.8 ± 0.4	12/12	6.8 ± 0.3
1	5/6	8.0 ± 1.0	5/5	8.6 ± 1.6
0.1	0/5	—	1/5	9.0 ± 0.0

^a^Mice were infected with the George’s disease 7 (GDVII) strain of Theiler’s murine encephalomyelitis virus (TMEV) intracerebrally.

^b^Plaque forming units (PFUs) of virus inoculated.

^c^Number of dead mice/total number of mice inoculated with virus.

^d^Mean survival days ± standard error of the mean (SEM) in dead mice following GDVII virus infection.

### Gata3 overexpression does not alter susceptibility to TMEV infection

Using Th2-biased Gata3-tg mice, we determined whether an increase in Th2 immune responses could alter susceptibility to DA virus infection. We infected wild-type mice and Gata3-tg mice on the C57BL/6 mouse background with DA virus and monitored the clinical signs for 2 months. During the acute phase, both groups had similar incidence of seizures [wild-type, 66% (19 of 29 mice); Gata3-tg, 60% (20 of 33 mice), *P* = 0.6, χ^2^ test] and Racine scale scores (mean maximum scores ± SEM in seized mice: wild-type, 5 ± 0; Gata3-tg, 5 ± 0). The clinical course, severity, and weight changes were comparable between wild-type mice and Gata3-tg mice during the 2 months observation period (Supplementary Fig. 3).

To determine whether Gata3 overexpression could affect susceptibility to TMEV-induced pure viral pathology, we infected wild-type mice and Gata3-tg mice with 0.1 to 100 PFUs of GDVII virus. Most mice developed encephalitic signs around 6 days p.i. and died within 10 days p.i. (Table 2). Both groups had similar fatality, survival periods, and LD_50_ titers (LD_50_ titers: wild-type, 0.87 PFUs; Gata3-tg, 0.74 PFUs).

**Table 2:** Mortality and survival days of wild-type mice and Gata3-tg mice in GDVII virus infection.

PFU^b^	Wild-type^a^		Gata3-tg	
	Mortality^c^	Survival days ± SEM^d^	Mortality	Survival days ± SEM
100	6/6	6.2 ± 0.2	5/5	6.8 ± 0.6
10	6/7	6.8 ± 0.3	5/5	6.6 ± 0.4
1	8/14	9.5 ± 0.7	4/8	8.3 ± 0.3
0.1	0/5	—	1/4	11 ± 0.0

^a^Mice were infected with the GDVII strain of TMEV intracerebrally.

^b^PFUs of virus inoculated.

^c^Number of dead mice/total number of mice inoculated with virus.

^d^Mean survival days ± SEM in dead mice following GDVII virus infection.

### Gata3-tg mice do not develop inflammatory demyelination in the CNS

**Figure 5:**
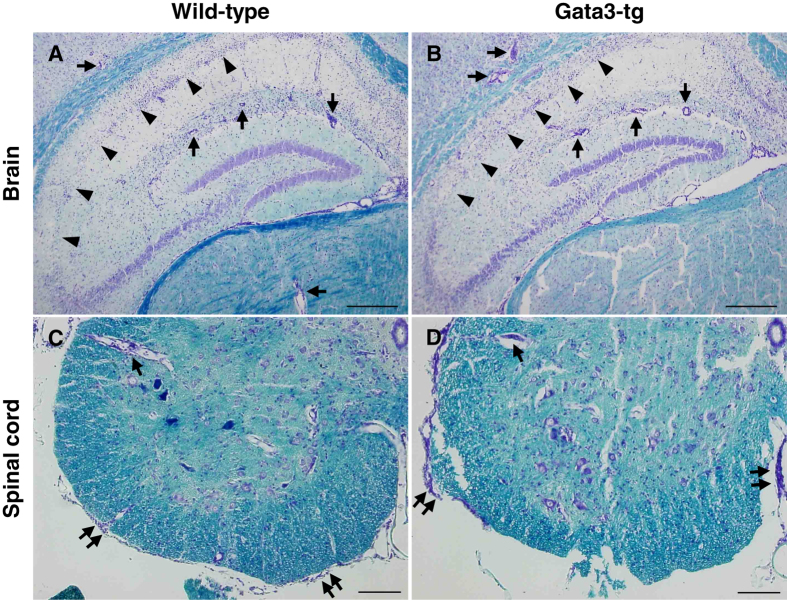
Gata3 overexpression did not alter neuropathology in TMEV infection. (**A**–**D**) Luxol fast blue stains of the CNS tissue sections from wild-type mice and Gata3-tg mice 1 week after DA virus infection. In the brain sections (scale bar = 300 μm), arrows and arrowheads show perivascular cuffing and neuronal loss, respectively. In the spinal cord sections (scale bar = 200 μm), paired arrows and arrows show meningitis and perivascular cuffing, respectively. Tissue sections are representative of two independent experiments.

Since we previously demonstrated that DA virus-infected RORγt-tg mice on the C57BL/6 mouse background developed inflammatory demyelination in the CNS with no clinical signs^48^, we compared the neuropathology between DA virus-infected wild-type mice and Gata3-tg mice. During the acute phase (1 week p.i.), both wild-type mice and Gata3-tg mice had severe neuronal loss with infiltration of inflammatory cells in the brain, particularly in the region CA1 (pyramidal cell layer) of the hippocampus (Fig. 5A,B). The brain inflammation scores were comparable between the two groups (Supplementary Fig. 4A). In the spinal cord, 1 week p.i., both wild-type mice and Gata3-tg mice developed meningitis and perivascular cuffing, but not demyelination, and had similar pathology scores (Fig. 5C,D and Supplementary Fig. 4B). During the chronic phase (2 months p.i.), both wild-type mice and Gata3-tg mice had similar hippocampal atrophy in the brain, which is shown by neuronal loss in the region CA1 (Supplementary Fig. 5A,B). In the spinal cord, both wild-type mice and Gata3-tg mice did not develop demyelinating lesions, 2 month p.i. (Supplementary Fig. 5C,D).

### Gata3-tg mice mount anti-viral cellular and humoral responses comparable to wild-type mice

**Figure 6:**
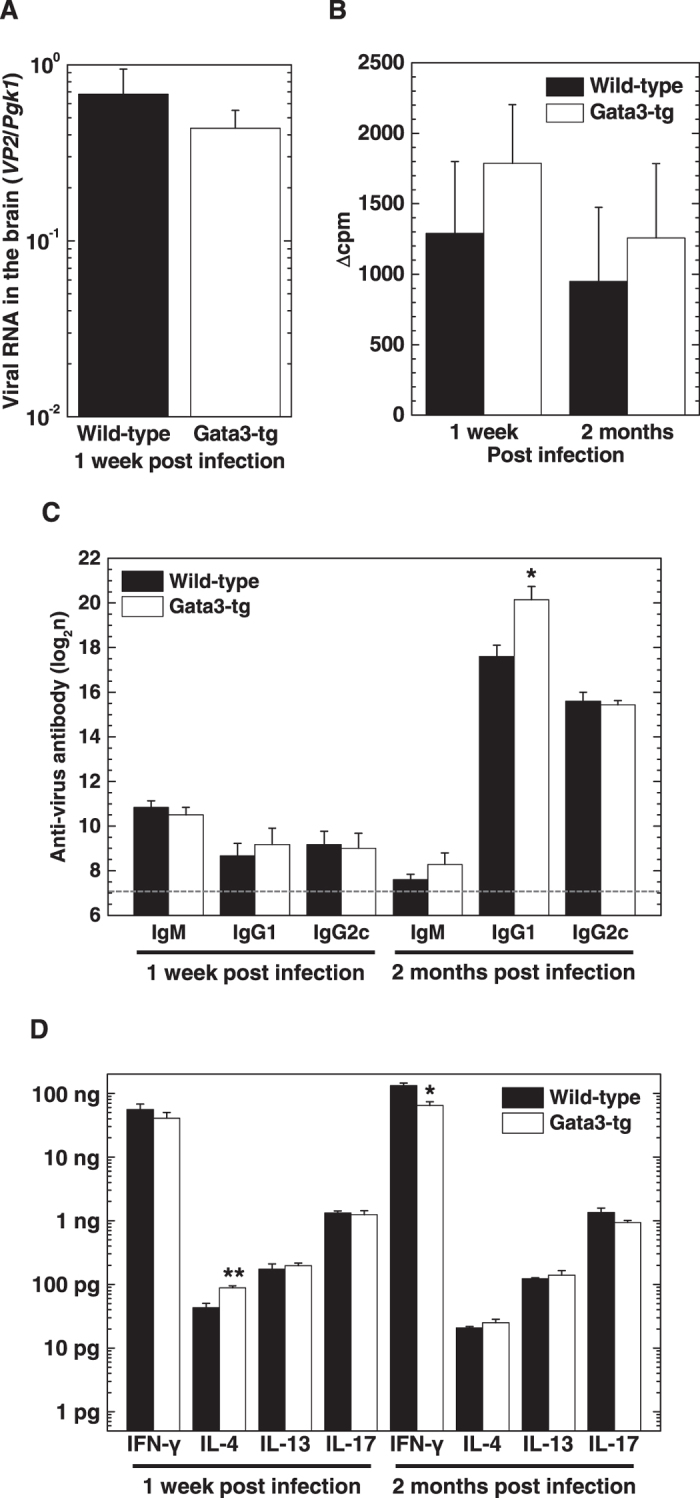
Gata3-tg mice and wild-type mice mounted similar anti-viral immune responses. (**A**) Real-time PCR analyses of the capsid protein *VP2* of TMEV in the brains from wild-type mice (black bar) and Gata3-tg mice (white bar) 1 week after DA virus infection. Values are the mean + SEM of five to eight mice. (**B**) TMEV-specific lymphoproliferative responses of splenic MNCs from wild-type mice and Gata3-tg mice 1 week and 2 months after DA virus infection. Values of lymphoproliferative responses to TMEV are expressed as Δcpm: (mean of experimental cpm in TMEV-specific stimulation) − (mean of control cpm). Values are the mean + SEM of three to four pools of spleens from two to three mice per time point. (**C**) ELISAs of anti-TMEV IgM, IgG1, and IgG2c subclasses in sera from wild-type mice and Gata3-tg mice 1 week and 2 months after DA virus infection. The dotted line shows the detection limit. Values are the mean + SEM of four to seven mice per group. (**D**) ELISAs of IFN-γ, IL-4, IL-13, and IL-17 production from mitogen-stimulated splenic MNCs of wild-type mice and Gata3-tg mice 1 week and 2 months after DA virus infection. Values are the mean + SEM of five to seven mice per time point. (**A**–**D**) The experiments were conducted twice independently. *P < 0.05 and **P < 0.01, Student *t* test.

Since Gata3-tg mice remained as resistant to TMEV infection during the acute and chronic phases as wild-type mice, we anticipated that viral clearance and anti-viral immune responses could also be similar between the two mouse strains. We found that the levels of viral RNA in the brain 1 week p.i. were similar between wild-type mice and Gata3-tg mice (Fig. 6A). Both wild-type mice and Gata3-tg mice eradicated the virus from the CNS 2–3 weeks p.i. (data not shown). During the acute phase of TMEV infection, resistant mouse strains have been shown to mount substantial cellular and humoral immune responses, both of which contribute to viral clearance^48^. We found that both wild-type mice and Gata3-tg mice mounted similar levels of anti-TMEV cellular immune responses by [^3^H]thymidine incorporation assays (Fig. 6B). Anti-TMEV antibody responses, including IgM, IgG1 and IgG2c, were also similar between wild-type mice and Gaga3-tg mice during the acute phase, while only anti-TMEV IgG1 titers were statistically higher in Gata3-tg mice during the chronic phase (Fig. 6C).

Using ELISAs, we also determined cytokine profiles in wild-type mice and Gata3-tg mice 1 week and 2 months p.i. The levels of IFN-γ production were lower in Gata3-tg mice (2 months p.i., *P* < 0.05), while the levels of IL-4 production were higher in Gata3-tg mice (1 week p.i., *P* < 0.01), compared with wild-type mice (Fig. 6D). On the other hand, there were no significant differences in the levels of IL-13 or IL-17 production between wild-type mice and Gata3-tg mice.

## Discussion

Clinically, immune system imbalance has been reported to be associated with susceptibility to human diseases. For example, Th1- or Th17-biased responses play a pathogenic role in autoimmune diseases, while Th2-biased responses contribute to the pathogenesis of allergic diseases^53^. Recently, there have been reports that patients with “gain-of-function” mutations in *STAT*1 gene had normal Th1 immune responses, but impaired Th17 immune responses, resulting in severe infections with viruses, including a herpesvirus and parapoxvirus, as well as fungi^21, 22^. In this study using T-bet-tg mice and Gata3-tg mice on the resistant C57BL/6 mouse background, we demonstrated that T-bet, but not Gata3, overexpression in T cells was detrimental in neurotropic TMEV infection. Despite being infected with a less virulent DA virus, most T-bet-tg mice developed a fatal disease with impaired viral clearance. Although TMEV-infected T-bet-tg mice had a Th1-biased cytokine profile, the reason why an expected increase in the Th1 responses was not seen in the T-bet-tg mice was likely due to severe depletion of T cells in the spleen in T-bet tg mice following TMEV infection. The T cell depletion can explain low anti-viral cellular immune responses, while low Th2/Th17 cytokine production that was also seen in the T-bet-tg mice could further result in the poor anti-viral antibody production.

“Loss-of-function” studies using neutralizing antibodies against Th1-associated cytokines have shown that Th1 immune responses play contrasting roles in TMEV infection. Pullen *et al*.^54^ demonstrated that treatment with IFN-γ-neutralizing monoclonal antibody (mAb) in TMEV-infected susceptible SJL/J mice accelerated the disease course and exacerbated the CNS pathology, compared with control mice, suggesting that Th1 cells play a protective role. On the other hand, Pullen *et al*.^54^ also showed that TMEV-infected mice with IFN-γ infusion intracerebrally showed clinical signs within 3 days of IFN-γ injection (8 days p.i.), suggesting a pathogenic role of Th1 cells. Furthermore, Inoue *et al*.^17^ demonstrated that TMEV-infected susceptible SJL/J mice with IL-12-neutralizing mAb treatment, which decreases Th1 cell differentiation, had a delay in the onset of TMEV-IDD with less severe inflammatory demyelination in the CNS, compared with control mice, suggesting that Th1 cells play a pathogenic role. In this study, we demonstrated that T-bet-tg mice infected with DA virus intracerebrally had Th1-biased responses and most mice died by 18 days p.i. Thus, T-bet overexpression seems to be detrimental in TMEV infection.

We demonstrated that DA virus-infected T-bet-tg mice had significantly lower anti-TMEV antibody titers, while DA virus-infected Gata3-tg mice had higher anti-TMEV IgG1 titers, compared with wild-type mice. These findings may be due to the difference in Th2 cytokine responses, which help antibody production; we found the ratios of Th1 (IFN-γ)/Th2 (IL-4) cytokine responses were higher in T-bet-tg mice and lower in Gata3-tg mice (data not shown). The differences of anti-TMEV antibody titers due to the different Th1/Th2 cytokine balance may affect viral clearance in the CNS. In addition, T-bet-tg mice had a significant decrease in IL-17 production. The decreased Th17 responses may also contribute to decreased anti-TMEV antibody responses, since Th17 cells have been shown to facilitate antibody production^55^. Since antibodies have been shown to be taken up by neurons^56^, antibodies could contribute to viral clearance from the CNS in neurotropic viral infections. In contrast, since neurons express neither MHC class I nor MHC class II molecules during inflammation as well as steady state^57, 58^, anti-viral T cells could not kill virus-infected neurons. For example, in Sindbis virus infection, passive transfer of anti-viral antibodies into severe combined immunodeficient (SCID) mice, which have neither functional T cells nor antibodies, resulted in viral clearance from the CNS, while adoptive transfer of virus-specific T cells into SCID mice had no effect^59^.

In TMEV infection, Fujinami *et al*.^60^ demonstrated that anti-TMEV mAb treatment of athymic nude mice, which cannot mount neither cellular nor humoral immunity, protected from the lethal infection with a significant reduction of infectious virus in the CNS. Similarly, Welsh *et al*.^61^ demonstrated that CD4^+^ T cell-depleted CBA mice failed to produce anti-TMEV antibodies and died by 3 weeks p.i. On the other hand, a lack of anti-TMEV antibody responses does not necessarily result in fatal encephalitis. TMEV-infected MHC class II-deficient mice on the C57BL/6 mouse background^62^ cannot produce anti-TMEV antibodies, which lead to viral persistence, but not fatal acute encephalitis^63^. TMEV-induced encephalitis was observed in B cell-deficient (μMT) C57BL/6 mice only if the mice depleted of CD8^+^ cells, while anti-TMEV serum transfer into anti-CD8 mAb-treated μMT mice restored resistance to encephalitis^44^. TMEV-infected β2-microglobulin mice lacking functional CD8^+^ T cells on the C57BL/6^46^ or C57BL/6 × 129^45, 47^ mouse background also failed to clear TMEV, leading to viral persistence, while these mice did not succumb to acute encephalitis. Taken together, TMEV-infected mice generally develop fatal acute encephalitis when the mice have neither antibody (which requires CD4^+^ T cell help) nor CD8^+^ T cell responses. This is consistent with fatal encephalitis seen in our TMEV-infected T-bet-tg mice whose anti-viral antibody and CD4^+^/CD8^+^ T cell responses were significantly decreased.

In this study, we demonstrated that TMEV infection triggered atrophy in the splenic white pulp due to depletion of T cells in T-bet-tg mice. Since TMEV cannot infect lymphocytes^64^, lymphoid depletion could be likely caused in a bystander fashion. Virus-induced lymphoid depletion has been observed in several viral infections, particularly viral hemorrhagic fevers (VHFs), where the virus infects macrophages and dendritic cells, but not lymphocytes, leading to lymphoid depletion^65^. The precise mechanism of the virus-induced lymphoid depletion in VHF is unclear, but is likely to depend on multiple pathways, including dysfunction of dendritic cells^66^. Since TMEV infects monocyte-macrophage lineage cells, dysfunction of macrophages and dendritic cells may also play a role in lymphoid depletion in TMEV-infected T-bet-tg mice.

TMEV-infected T-bet-tg mice had a significant reduction of *CD4*, *CD8a*, *Ifng*, and *Gzmb*, but not *Nkp46*, expression in the brain with decreased anti-TMEV cellular responses; following TMEV infection, T-bet-tg mice had impaired acquired immunity but not innate immunity, which was associated with splenic T cell depletion. The impaired TMEV-specific lymphoproliferation in TMEV-infected T-bet-tg mice would result in a significant decrease in anti-TMEV antibody titers, since Th cells have been shown to contribute to antibody production in the white pulp^61^. Consistent with these findings, Kondo *et al*.^28^ demonstrated that T-bet-tg mice became resistant to a CIA model with lower titers of antibody against type II collagen. The absolute number of Th cells in the spleen tended to be lower in T-bet-tg mice than in wild-type mice after immunization. Furthermore, naïve T-bet-tg mice had a significant decrease in the absolute number of thymocytes, compared with wild-type mice. These findings suggest that T-bet-tg mice may be immunocompromised and that viral pathology may cause fatal infection in T-bet-tg mice, since T-bet-tg mice had failure of viral clearance associated with impaired acquired immune responses to DA virus with T cell depletion in the spleen.

During the first week of TMEV infection, C57BL/6 mice develop seizures; anti-viral innate immunity, but not acquired immunity, has been shown to play a pathogenic role in TMEV-induced seizures^67–69^. In this study, there were no statistical differences in the incidence of DA virus-induced seizures between wild-type mice versus T-bet-tg mice and Gata3-tg mice. Wild-type mice and the two tg mice had seizures from days 3 to 8 after DA virus infection mainly, when innate immunity plays a role in viral clearance. Furthermore, no statistical differences were seen in the levels of *Nkp46*, *Ifng* or *Gzmb* in the brain 4 days p.i. between wild-type mice versus T-bet-tg mice. These findings would be reasonable, since T-bet or Gata3 overexpression in T cells in T-bet-tg mice or Gata3-tg mice unlikely affects innate immune responses.

Neurovirulent GDVII virus causes fatal acute polioencephalomyelitis regardless of mouse strains: infected mice have severe weight loss and encephalitic signs, including hunched back and ruffled fur, and die within 10 days of the infection^70^. Viral pathology has been shown to cause the fatal outcome here, since infected mice failed to induce anti-viral acquired immune responses^34^. In this study, we demonstrated that the survival periods and LD_50_ titers were similar between wild-type mice versus T-bet-tg mice and Gata3-tg mice. These results could be reasonable in GDVII virus infection, which is a pure viral pathology model^34^.

In summary, we demonstrated that T-bet overexpression in T cells was detrimental in TMEV infection due to impaired acquired immune responses to TMEV, which were associated with atrophy of the spleen. On the other hand, Gata3 overexpression in T cells had some beneficial effects on TMEV infection by increasing anti-viral IgG1 production with a Th2-biased cytokine profile. Thus, an individual, who has overexpression of T-bet, may be more susceptible to viral infections, while an individual, who has overexpression of Gata3, may be resistant to viral infections.

## Methods

### Animal experiments

To generate T-bet-tg mice or Gata3-tg mice, a full-length cDNA encoding the murine T-bet or Gata3 protein was inserted into a VA CD2 transgene cassette that contained the upstream gene regulatory region and locus control region of the human *CD2* gene^26, 71^. T-bet-tg mice and Gata3-tg mice preferentially overexpress T-bet and Gata3 in T cells, respectively. T-bet-tg mice and Gata3-tg mice were maintained as heterozygotes for the transgene by being bred with wild-type C57BL/6 mice^24^. The mice were maintained under specific pathogen-free conditions in our animal care facility at Louisiana State University Health Sciences Center-Shreveport (LSUHSC-S) and Kindai University Faculty of Medicine (Osaka, Japan). All experimental procedures were reviewed and approved by the Institutional Animal Care and Use Committee of LSUHSC-S and Kindai University Faculty of Medicine, and performed according to the criteria outlined by the National Institutes of Health (NIH).

Six to eight-week-old wild-type mice (littermate controls), T-bet-tg mice, and Gata3-tg mice were infected intracerebrally with 2 × 10^5^ PFUs of DA virus or 0.1 to 100 PFU of GDVII virus^70^. Clinical signs of seizures were evaluated using the Racine scale: 1, mouth and facial movements; 2, head nodding; 3, forelimb clonus; 4, rearing; and 5, rearing and falling^50, 51^. The LD_50_ titers were calculated using the Reed and Muench calculation of the 50% endpoint^72^.

### Real-time PCR

Following perfusion with phosphate-buffered saline (PBS) into the heart, the brain was harvested, frozen with liquid nitrogen, and then homogenized with TRI-reagent (Molecular Research Center, Inc., Cincinnati, OH) using a Polytron PT1200E homogenizer (Kinematica AG, Luzern, Switzerland)^35^. RNA was isolated from the homogenate using a Qiagen RNeasy Mini Kit (Qiagen, Inc., Valencia, CA), according to the manufacturer’s instruction. We reverse-transcribed 1 µg of total RNA into cDNA using the ImProm-II^TM^ Reverse Transcription System (Promega, Corp. Madison, WI)^73^. Using 50 ng of cDNA, real-time PCR was conducted with an RT^2^ Fast SYBR Green/Flurescein qPCR Master Kit (Qiagen) and the MyiQ^TM^2 Real Time PCR Detection System (Bio-Rad Laboratories, Inc., Hercules, CA). To determine viral replication and gene expression related to CD4^+^ T cells, CD8^+^ T cells, and NK cells in the brain, we used the following primer pairs: the capsid protein *VP2* of TMEV (Real Time Primers, LLC, Elkins Park, PA), forward (5′-TGGTCGACTCTGTGGTTACG-3′) and reverse (5′-GCCGGTCTTGCAAAGATAGT-3′)^74^; *Cd4* (Eurofins Genomics, Tokyo, Japan), forward (5′-TCCTAGCTGTCACTCAAGGGA-3′) and reverse (5′-TCAGAGAACTTCCAGGTGAAGA-3′); *Cd8a* (Eurofins Genomics), forward (5′-TGCTGTCCTTGATCATCACTCT-3′) and reverse (5′-ACTAGCGGCCTGGGACAT-3′); *Ifng* (Real Time Primers), forward (5′-CAAAAGGATGGTGACATGAA-3′) and reverse (5′-TTGGCAATACTCATGAATGC-3′); *Gzmb* (Real Time Primers), forward (5′-TGGCCTTACTTTCGATCA AG-3′) and reverse (5′-CAGCATGATGTCATTGGAGA-3′); and *Nkp46* (Eurofins Genomics), forward (5′-ATGCTGCCAACACTCACTG-3′) and reverse (5′-GATGTTCACCGAGTTTCCATTTG-3′). A primer pair for *phosphoglycerate kinase 1* (*Pgk1*) (Real Time Primers) was used as a housekeeping gene for normalization; forward (5′-GCAGATTGTTTGGAATGGTC-3^′^) and reverse (5′-TGCTCACATGGCTGACTTTA-3′).

### Lymphoproliferative responses to TMEV

Lymphoproliferation was assessed as described previously^48^. We harvested the spleen and isolated MNCs from the spleen pools of two to three TMEV-infected mice using Histopaque®-1083 (Sigma-Aldrich, St. Louis, MO). Splenic MNCs were cultured with RPMI1640 medium (Mediatech, Inc., Manassas, VA) supplementing 10% fetal bovine serum (FBS) (Mediatech), 2 mM L-glutamine (Mediatech), 50 mM β-mercaptoethanol (Sigma-Aldrich), and 1% antibiotic-antimycotic solution (Mediatech), at 2 × 10^5^ cells/well in 96-well plates (Corning Inc., Corning, NY) and stimulated with 2 × 10^5^ cells/well of TMEV-infected antigen-presenting cells (TMEV-APCs) or mock-infected antigen-presenting cells (control-APCs) for 5 days. TMEV-APCs were made from whole spleen cells infected *in vitro* with TMEV at a multiplicity of infection (MOI) of 1, while control-APCs were made from mock-infected whole spleen cells. Both TMEV-APCs and control-APCs were incubated overnight and irradiated with 2,000 rads using a ^137^Cs irradiator (J.L. Shepherd & Associates, San Fernando, CA). To assess the levels of lymphoproliferative responses, [^3^H]thymidine (PerkinElmer, Inc., Waltham, MA) was added in the culture system at the concentration of 1 μCi/well for the last 24 hours. The incorporated radioactivity was measured using a Wallac 1409 Liquid Scintillation Counter (PerkinElmer). All cultures were performed in triplicate and the data were expressed as Δcpm: (mean of cpm in TMEV-APC stimulation) − (mean of cpm in control-APC stimulation).

### ELISAs for anti-TMEV antibodies and cytokines

The titers of serum anti-TMEV antibodies were quantified by ELISAs, as described previously^41^. Blood was drawn from the heart and spun down for harvesting serum. We coated 96-well flat-bottom Nunc-Immuno plates (Thermo Fisher Scientific Inc., Waltham, MA) with 10 μg/ml of TMEV antigen. Eleven serial two-fold dilutions from 2^7^ of serum were added to the plates followed by a peroxidase-conjugated anti-mouse IgM (Stressgen Bioreagents Corp., Victoria, Canada), IgG1 (Thermo Fisher Scientific), or IgG2c antibody (Thermo Fisher Scientific). Immunoreactive complexes were detected with *o*-phenylendiamine dihydrochloride (Sigma-Aldrich). The absorbances were read at 492 nm using a Multiskan MCC/340 Microplate Reader (Thermo Fisher Scientific). An absorbance higher than the mean + two standard deviations of naïve serum samples at a dilution of 2^7^ was used as the standard for evaluating anti-TMEV antibody titers^48^.

Cytokine ELISAs were conducted as described previously^39^. MNCs from the spleen pools of two TMEV-infected mice were cultured at 8 × 10^6^ cells/well in 6-well plates (Corning) and stimulated with 5 μg/ml of concanavalin A for 48 hours. Culture supernatants were harvested and stored at −80 °C until examined. The amounts of IFN-γ (BD Biosciences, San Diego, CA), IL-4 (BD Biosciences), IL-13 (Thermo Fisher Scientific), and IL-17A (BioLegend, San Diego, CA) production in the culture supernatants were quantified using the ELISA kits, according to the manufacturer’s instructions.

### Histology and immunohistochemistry

TMEV-infected mice were perfused with PBS followed by a 4% paraformaldehyde (PFA, Sigma-Aldrich) solution in PBS^75^. After the PFA fixation, the spleen was harvested from mice and embedded in paraffin. The spleen tissues were sliced at 4 μm-thick using an HM 325 Rotary Microtome (Thermo Fisher Scientific) and stained with hematoxylin (Electron Microscopy Sciences, Hatfield, PA) and eosin (Thermo Fisher Scientific) to visualize the general architecture. T cells, B cells, and macrophages in spleen sections were visualized by immunohistochemistry with anti-CD3 antibody (Biocare Medical, Pacheco, CA), anti-B220 antibody (Bio-Rad Laboratories), and anti-F4/80 antibody (eBiosciences, San Diego, CA), respectively, using a Histofine MAX-PO kit (Nichirei Biosciences, Tokyo, Japan). Antigen retrieval was conducted by citrate buffer (pH 6, Dako, Carpinteria, CA) and proteinase K (AMRESCO LLC, Solon, OH) prior to incubation with antibodies to CD3 and F4/80, respectively.

After the PFA fixation, the brain and spinal cord were divided into five coronal slabs and 10 to 12 transversal segments, respectively, and embedded in paraffin. The CNS tissues were sliced at 4 μm-thick and stained with Luxol fast blue (Solvent blue 38; Sigma-Aldrich) for myelin visualization. Histological scoring of the CNS was performed as described previously^39^. A brain pathology score was determined by combining pathology scores of meningitis, perivascular cuffing, and demyelination. These scores were evaluated as follows: meningitis: 0, no meningitis; 1, mild cellular infiltration; 2, moderate cellular infiltration; and 3, severe cellular infiltration; perivascular cuffing: 0, no cuffing; 1, 1 to 10 lesions; 2, 11 to 20 lesions; 3, 21 to 30 lesions; 4, 31 to 40 lesions; and 5, over 40 lesions; demyelination: 0, no demyelination; 1, mild demyelination; 2, moderate demyelination; and 3, severe demyelination. For scoring of spinal cord sections, each spinal cord section was divided into four quadrants: the ventral funiculus, the dorsal funiculus, and each lateral funiculus. Any quadrant containing meningitis, perivascular cuffing, or demyelination was given a score of 1 in that pathological class. The total number of positive quadrants for each pathological class was determined and then divided by the total number of quadrants present on the slide and multiplied by 100 to give the percent involvement for each pathological class. An overall pathology score was also determined by giving a positive score if any pathology was seen in the quadrant, and presented as the percent involvement.

### Statistical analysis

Using the OriginPro 8.1 (OriginLab Corporation, Northampton, MA), the Student *t* test and Mann-Whitney *U* test were conducted for parametric data and nonparametric data, respectively. The χ^2^ test was conducted for categorical data using the GraphPad software (GraphPad Software, Inc., La Jolla, CA).

## Electronic supplementary material

Supplementary Information

## References

[1] Mosmann TR, Cherwinski H, Bond MW, Giedlin MA, Coffman RL (1986). Two types of murine helper T cell clone. I. Definition according to profiles of lymphokine activities and secreted proteins. J Immunol.

[2] Bettelli E, Oukka M, Kuchroo VK (2007). T_H_-17 cells in the circle of immunity and autoimmunity. Nat Immunol.

[3] Wilson CB, Rowell E, Sekimata M (2009). Epigenetic control of T-helper-cell differentiation. Nat Rev Immunol.

[4] Szabo SJ (2000). A novel transcription factor, T-bet, directs Th1 lineage commitment. Cell.

[5] Szabo SJ (2002). Distinct effects of T-bet in T_H_1 lineage commitment and IFN-γ production in CD4 and CD8 T cells. Science.

[6] Zheng W-P, Flavell RA (1997). The transcription factor GATA-3 is necessary and sufficient for Th2 cytokine gene expression in CD4 T cells. Cell.

[7] Pai S-Y, Truitt ML, Ho I-C (2004). GATA-3 deficiency abrogates the development and maintenance of T helper type 2 cells. Proc Natl Acad Sci USA.

[8] Zhou L (2008). TGF-β-induced Foxp3 inhibits T_H_17 cell differentiation by antagonizing RORγt function. Nature.

[9] Sabet-Baktach M (2013). Double deficiency for RORγt and T-bet drives Th2-mediated allograft rejection in mice. J Immunol.

[10] Bettelli E (2006). Reciprocal developmental pathways for the generation of pathogenic effector T_H_17 and regulatory T cells. Nature.

[11] Lebrun A (2015). T-bet Is Required for the Rapid Clearance of Attenuated Rabies Virus from Central Nervous System Tissue. J Immunol.

[12] Maloy KJ (2000). CD4^+^ T cell subsets during virus infection: protective capacity depends on effector cytokine secretion and on migratory capability. J Exp Med.

[13] Matsui M, Moriya O, Yoshimoto T, Akatsuka T (2005). T-bet is required for protection against vaccinia virus infection. J Virol.

[14] Swain SL, McKinstry KK, Strutt TM (2012). Expanding roles for CD4^+^ T cells in immunity to viruses. Nat Rev Immunol.

[15] Martinez NE (2013). Immunopathological patterns from EAE and Theiler’s virus infection: Is multiple sclerosis a homogenous 1-stage or heterogenous 2-stage disease?. Pathophysiology.

[16] Rouse BT, Sehrawat S (2010). Immunity and immunopathology to viruses: what decides the outcome?. Nat Rev Immunol.

[17] Inoue A (1998). Suppressive effect on Theiler’s murine encephalomyelitis virus-induced demyelinating disease by the administration of anti-IL-12 antibody. J Immunol.

[18] van den Broek M (2000). IL-4 and IL-10 antagonize IL-12-mediated protection against acute vaccinia virus infection with a limited role of IFN-γ and nitric oxide synthetase 2. J Immunol.

[19] Uzel G (2013). Dominant gain-of-function *STAT1* mutations in *FOXP3* wild-type immune dysregulation-polyendocrinopathy-enteropathy-X-linked-like syndrome. J Allergy Clin Immunol.

[20] Takezaki S (2012). Chronic mucocutaneous candidiasis caused by a gain-of-function mutation in the STAT1 DNA-binding domain. J Immunol.

[21] Tóth B (2012). Herpes in *STAT1* gain-of-function mutation. Lancet.

[22] Kilic SS, Puel A, Casanova J-L (2015). Orf infection in a patient with STAT1 gain-of-function. J Clin Immunol.

[23] Kiwamoto T (2006). Transcription factors T-bet and GATA-3 regulate development of airway remodeling. Am J Respir Crit Care Med.

[24] Martinez NE (2014). RORγt, but not T-bet, overexpression exacerbates an autoimmune model for multiple sclerosis. J Neuroimmunol.

[25] Fernando V (2014). Regulation of an autoimmune model for multiple sclerosis in Th2-biased GATA3 transgenic mice. Int J Mol Sci.

[26] Ishizaki K (2007). Th1 and type 1 cytotoxic T cells dominate responses in T-bet overexpression transgenic mice that develop contact dermatitis. J Immunol.

[27] Shimohata H (2009). Overexpression of T-bet in T cells accelerates autoimmune glomerulonephritis in mice with a dominant Th1 background. J Nephrol.

[28] Kondo Y (2012). Overexpression of T-bet gene regulates murine autoimmune arthritis. Arthritis and rheumatism.

[29] Kimura T (2006). Overexpression of the transcription factor GATA-3 enhances the development of pulmonary fibrosis. Am J Pathol.

[30] Ano S (2013). Transcription factors GATA-3 and RORγt are important for determining the phenotype of allergic airway inflammation in a murine model of asthma. J Immunol.

[31] Theiler M, Gard S (1940). Encephalomyelitis of mice. I. Characteristics and pathogenesis of the virus. J Exp Med.

[32] Lipton HL (1975). Theiler’s virus infection in mice: an unusual biphasic disease process leading to demyelination. Infection and immunity.

[33] Sato F (2013). Resveratrol exacerbates both autoimmune and viral models of multiple sclerosis. The American journal of pathology.

[34] Tsunoda I, Iwasaki Y, Terunuma H, Sako K, Ohara Y (1996). A comparative study of acute and chronic diseases induced by two subgroups of Theiler’s murine encephalomyelitis virus. Acta neuropathologica.

[35] Kawai E (2015). Organ-specific protective role of NKT cells in virus-induced inflammatory demyelination and myocarditis depends on mouse strain. J Neuroimmunol.

[36] Daniels JB, Pappenheimer AM, Richardson S (1952). Observations on encephalomyelitis of mice (DA strain). J Exp Med.

[37] Denic A (2011). The relevance of animal models in multiple sclerosis research. Pathophysiology.

[38] Sato F, Tanaka H, Hasanovic F, Tsunoda I (2011). Theiler’s virus infection: pathophysiology of demyelination and neurodegeneration. Pathophysiology.

[39] Martinez NE (2014). Protective and detrimental roles for regulatory T cells in a viral model for multiple sclerosis. Brain pathology.

[40] Tsunoda I, Tanaka T, Terry EJ, Fujinami RS (2007). Contrasting roles for axonal degeneration in an autoimmune versus viral model of multiple sclerosis: When can axonal injury be beneficial?. Am J Pathol.

[41] Sato F (2014). Distinct kinetics of viral replication, T cell infiltration, and fibrosis in three phases of myocarditis following Theiler’s virus infection. Cell Immunol.

[42] Pullen LC, Park SH, Miller SD, Dal Canto MC, Kim BS (1995). Treatment with bacterial LPS renders genetically resistant C57BL/6 mice susceptible to Theiler’s virus-induced demyelinating disease. J Immunol.

[43] Richards MH (2011). Virus expanded regulatory T cells control disease severity in the Theiler’s virus mouse model of MS. J Autoimmun.

[44] Kang B-S, Palma JP, Lyman MA, Dal Canto M, Kim BS (2005). Antibody response is required for protection from Theiler’s virus-induced encephalitis in C57BL/6 mice in the absence of CD8^+^ T cells. Virology.

[45] Fiette L, Aubert C, Brahic M, Rossi CP (1993). Theiler’s virus infection of β_2_-microglobulin-deficient mice. J Virol.

[46] Pullen LC, Miller SD, Dal Canto MC, Kim BS (1993). Class I-deficient resistant mice intracerebrally inoculated with Theiler’s virus show an increased T cell response to viral antigens and susceptibility to demyelination. Eur J Immunol.

[47] Rodriguez M (1993). Abrogation of resistance to Theiler’s virus-induced demyelination in H-2^b^ mice deficient in β2-microglobulin. J Immunol.

[48] Martinez NE (2015). Th17-biased RORγt transgenic mice become susceptible to a viral model for multiple sclerosis. Brain Behav Immun.

[49] Libbey JE (2008). Seizures following picornavirus infection. Epilepsia.

[50] Racine RJ (1972). Modification of seizure activity by electrical stimulation. II. Motor seizure. Electroencephalogr Clin Neurophysiol.

[51] Benkovic SA, O’Callaghan JP, Miller DB (2004). Sensitive indicators of injury reveal hippocampal damage in C57BL/6J mice treated with kainic acid in the absence of tonic-clonic seizures. Brain Res.

[52] Intlekofer AM (2005). Effector and memory CD8^+^ T cell fate coupled by T-bet and eomesodermin. Nat Immunol.

[53] Zhu J, Paul WE (2008). CD4 T cells: fates, functions, and faults. Blood.

[54] Pullen LC, Miller SD, Dal Canto MC, van der Meide PH, Kim BS (1994). Alteration in the level of interferon-γ results in acceleration of Theiler’s virus-induced demyelinating disease. J Neuroimmunol.

[55] Mitsdoerffer M (2010). Proinflammatory T helper type 17 cells are effective B-cell helpers. Proc Natl Acad Sci USA.

[56] Greenlee JE (2010). Purkinje cell death after uptake of anti-Yo antibodies in cerebellar slice cultures. J Neuropathol Exp Neurol.

[57] Joly E, Oldstone MBA (1992). Neuronal cells are deficient in loading peptides onto MHC class I molecules. Neuron.

[58] Pender, M. P. *An introduction to neuroimmunology*. In: Autoimmune neurological disease (eds Pender MP, McCombe PA). *Cambridge University Press* pp. 14–25 (1995).

[59] Levine B (1991). Antibody-mediated clearance of alphavirus infection from neurons. Science.

[60] Fujinami RS, Rosenthal A, Lampert PW, Zurbriggen A, Yamada M (1989). Survival of athymic (*nu/nu*) mice after Theiler’s murine encephalomyelitis virus infection by passive administration of neutralizing monoclonal antibody. J Virol.

[61] Welsh CJR, Tonks P, Nash AA, Blakemore WF (1987). The effect of L3T4 T cell depletion on the pathogenesis of Theiler’s murine encephalomyelitis virus infection in CBA mice. J Gen Virol.

[62] Cosgrove D (1991). Mice lacking MHC class II molecules. Cell.

[63] Njenga MK (1996). Theiler’s virus persistence and demyelination in major histocompatibility complex class II-deficient mice. J Virol.

[64] McCright IJ, Fujinami RS (1997). Lack of correlation of Theiler’s virus binding to cells with infection. J Neurovirol.

[65] Zaki, S. R. & Peters, C. J. *Viral hemorrhagic fevers*. In: Pathology of infectious diseases (eds Connor DH, Chandler FW, Schwartz DA, Manz HJ, Lack EE). *Appleton and Lange* pp. 347–364 (1997).

[66] Geisbert TW, Jahrling PB (2004). Exotic emerging viral diseases: progress and challenges. Nat Med.

[67] Kirkman NJ, Libbey JE, Wilcox KS, White HS, Fujinami RS (2010). Innate but not adaptive immune responses contribute to behavioral seizures following viral infection. Epilepsia.

[68] Libbey JE, Kennett NJ, Wilcox KS, White HS, Fujinami RS (2011). Interleukin-6, produced by resident cells of the central nervous system and infiltrating cells, contributes to the development of seizures following viral infection. J Virol.

[69] Cusick MF, Libbey JE, Patel DC, Doty DJ, Fujinami RS (2013). Infiltrating macrophages are key to the development of seizures following virus infection. J Virol.

[70] Tsunoda I, Tanaka T, Fujinami RS (2008). Regulatory role of CD1d in neurotropic virus infection. J Virol.

[71] Matsuno Y (2007). Overexpression of GATA-3 protects against the development of hypersensitivity pneumonitis. Am J Respir Crit Care Med.

[72] Burleson, F. G., Chambers, T. M. & Wiedbrauk, D. L. Introduction to quantal virus assay. In: Virology: a laboratory manual (eds Burgleson FG, Chambers TM, Wiedbrauk DL). *Academic Press, Inc.* pp. 53–57 (1992)

[73] Omura S (2014). Bioinformatics multivariate analysis determined a set of phase-specific biomarker candidates in a novel mouse model for viral myocarditis. Circ Cardiovasc Genet.

[74] Deb C (2009). Demyelinated axons and motor function are protected by genetic deletion of perforin in a mouse model of multiple sclerosis. J Neuropathol Exp Neurol.

[75] Carlson, N. G., Hill, K. E., Tsunoda, I., Fujinami, R. S. & Rose, J. W. The pathologic role for COX-2 in apoptotic oligodendrocytes in virus induced demyelinating disease: implications for multiple sclerosis. *J Neuroimmunol* **174**, 21–31 (2006). 10.1016/j.jneuroim.2006.01.008 16516308

