# Supplementary material for: T-bet, but not Gata3, overexpression is detrimental in a neurotropic viral infection
Source: Sci Rep. 2017 Sep 5;7:10496. doi: 10.1038/s41598-017-10980-0 (PMC5585213; doi:10.1038/s41598-017-10980-0)
Supplement: Supplementary file 1 — Supplementary Information [file 41598_2017_10980_MOESM1_ESM.pdf]

Supplementary information

T-bet, but not Gata3, overexpression is detrimental in a neurotropic viral infection

Fumitaka Sato, Ph.D.,[a-d,#] Eiichiro Kawai, M.D., Ph.D.,[b,c,#] Nicholas E. Martinez, Ph.D., M.B.A.[b,c]
Seiichi Omura, Ph.D.,[a-d] Ah-Mee Park, Ph.D.,[a] Satoru Takahashi, M.D., Ph.D.,[e-h]
Keigyou Yoh, M.D., Ph.D,[e] and Ikuo Tsunoda, M.D., Ph.D.[a-d,*]

[a] Department of Microbiology
Kindai University Faculty of Medicine
377-2 Ohnohigashi, Osakasayama, Osaka 589-8511, Japan

[b] Department of Microbiology and Immunology
[c] Center for Molecular and Tumor Virology (CMTV)
[d] Center for Cardiovascular Diseases and Sciences (CCDS)
Louisiana State University Health Sciences Center-Shreveport (LSUHSC-S)
1501 Kings Highway, Shreveport, LA 71130, U.S.A.

[e] Department of Anatomy and Embryology, Faculty of Medicine
[f] International Institute for Investigative Sleep Medicine (WPI-IIIS)
[g] Life Science Center, Tsukuba Research Alliance (TARA)
[h] Laboratory Animal Resource Center (LARC)
University of Tsukuba
1-1-1 Tennodai, Tsukuba, Ibaraki 305-8575, Japan

[#] Drs. Sato and Kawai contributed equally.

* Corresponding author: Ikuo Tsunoda, M.D., Ph.D.
Department of Microbiology
Kindai University Faculty of Medicine
377-2 Ohnohigashi, Osakasayama, Osaka 589-8511, Japan
Tel: +81-72-366-0221
E-mail: itsunoda@med.kindai.ac.jp

Supplementary Figure 1

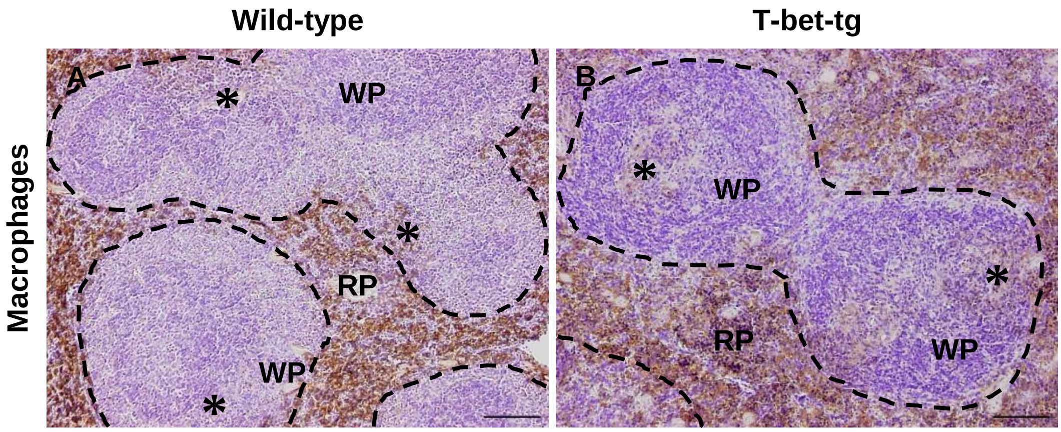

Supplementary Figure 1. Splenic red pulp (RP) immunostained for a macrophage-specific marker, F4/80. (**A, B**) No significant differences were seen in F4/80 staining between DA virus-infected wild-type (**A**) and T-bet-transgenic (tg) mice (**B**). The dotted lines show the white pulp (WP) and asterisks (*) are central arteries. RP, red pulp; WP, white pulp. Scale bar = 100 μm.

Supplementary Figure 2

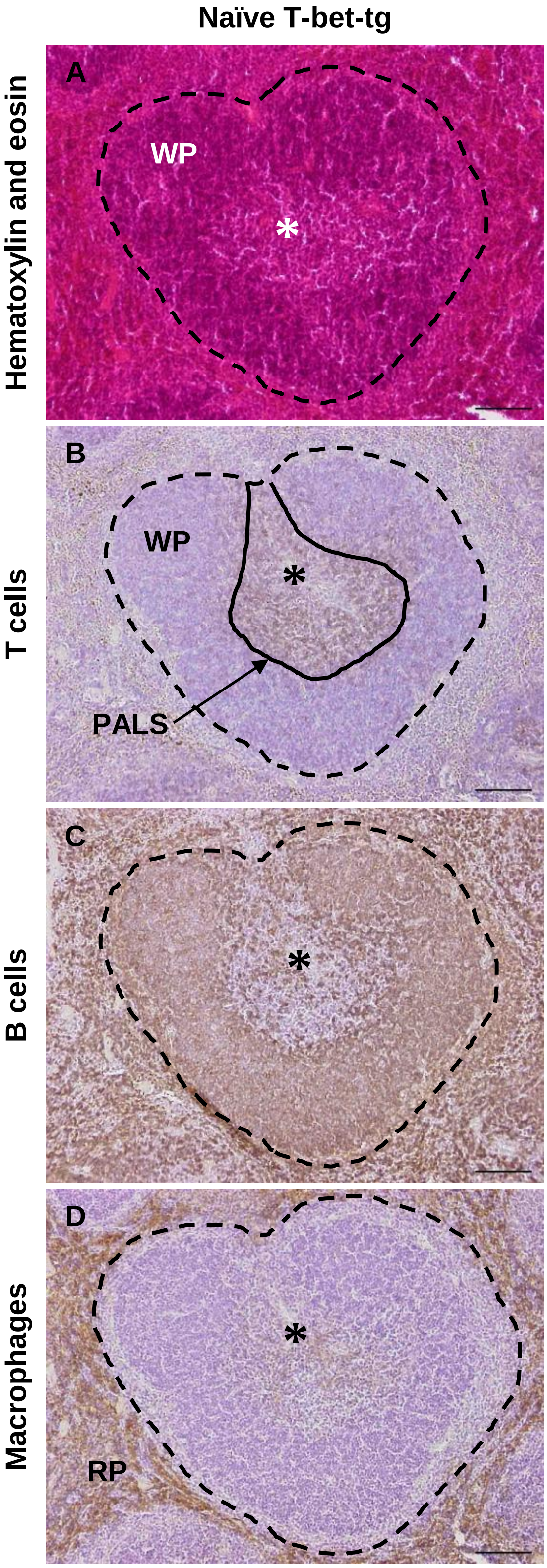

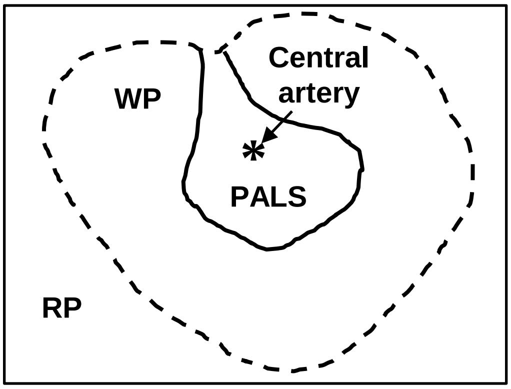

Supplementary Figure 2. Naïve T-bet-tg mice had normal splenic immune-architecture. (**A**) Hematoxylin and eosin staining. White pulp (WP, dotted line) with the central artery (*). (**B**) Immunohistochemistry against CD3 showed T cells in the periarterial lymphoid sheath (PALS, solid line). (**C, D**) Immunohistochemistry against B220 (**C**) and F4/80 (**D**) showed that B cells and macrophages were mainly seen in the white pulp and the red pulp (RP), respectively. Scale bar = 100 μm.

Supplementary Figure 3

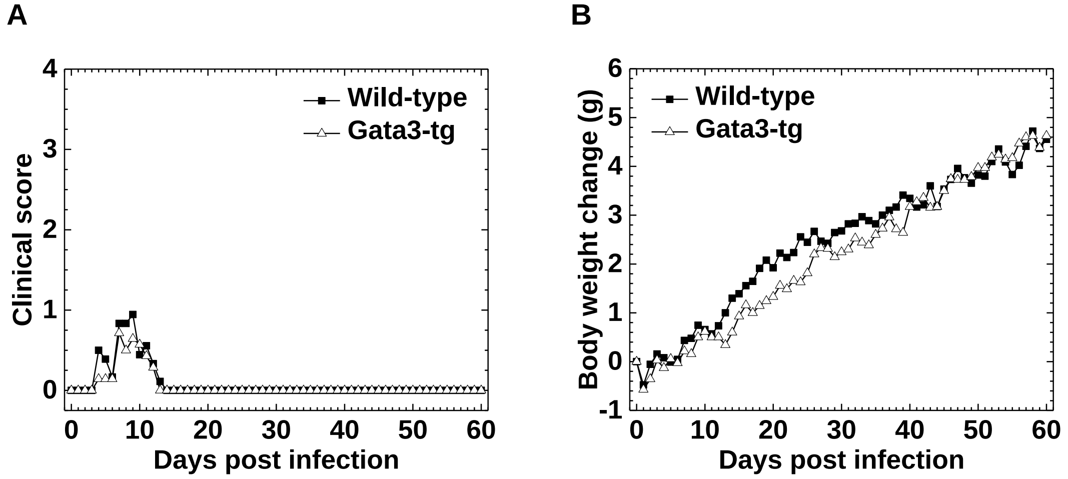

Supplementary Figure 3. Gata3 overexpression does not confer susceptibility to Theiler's murine encephalomyelitis virus (TMEV) infection. (**A**) Clinical scores of wild-type mice (closed boxes) and Gata3-tg mice (open triangles) 2 months after infection with the Daniels (DA) strain of TMEV (DA virus). Clinical scores were evaluated by measuring impaired righting reflexes: the proximal end of the mouse's tail was grasped and twisted to the right and then to the left (0, a healthy mouse resists being turn over; 1, the mouse is flipped onto its back but immediately rights itself on one side; 1.5, the mouse is flipped onto its back but immediately rights itself on one sides; 2, the mouse rights itself in 1 to 5 seconds; 3, righting takes more than 5 seconds; and 4, the mouse cannot right itself). (**B**) Body weight changes of DA virus-infected wild-type mice and Gata3-tg mice during the 2-months observation period. (**A, B**) Values are the mean of nine wild-type mice and seven Gata3-tg mice and representative of two independent experiments.

Supplementary Figure 4

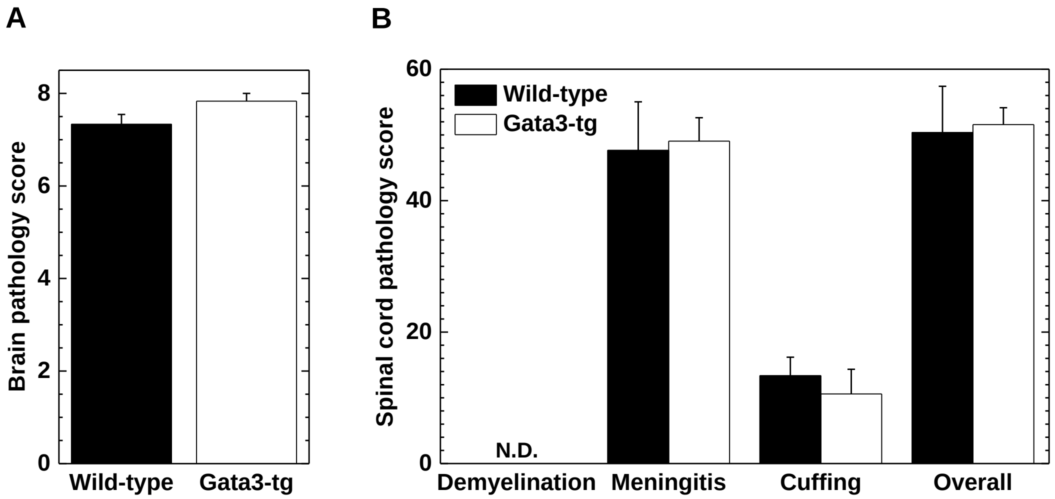

Supplementary Figure 4. Gata3 overexpression does not alter neuropathology scores during the acute phase of TMEV infection. (**A, B**) Brain and spinal cord pathology scores of wild-type mice (black bar) and Gata3-tg mice (white bar) 1 week after DA virus infection. N.D., not detectable. Values are the mean + standard error of the mean (SEM) of six mice per group. The experiments were conducted twice independently.

Supplementary Figure 5

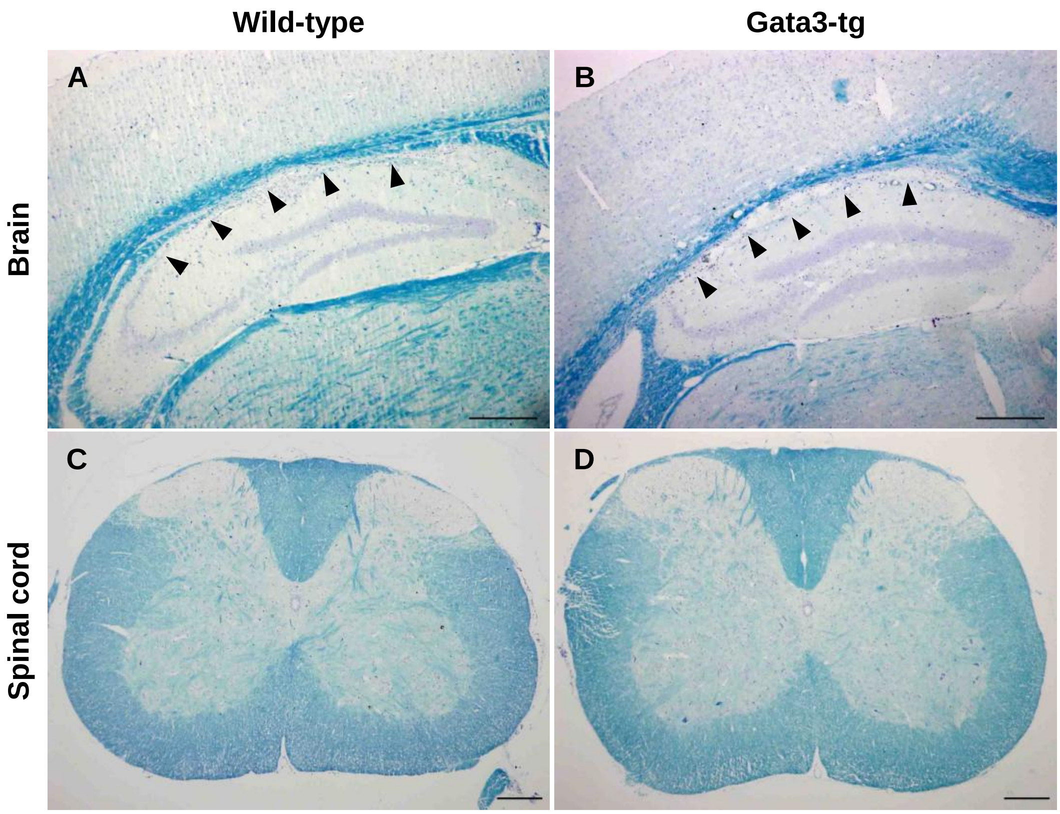

Supplementary Figure 5. Gata3 overexpression does not affect neuropathology during the chronic phase of TMEV infection. (**A-D**) Luxol fast blue stains of the brain and spinal cord sections from wild-type mice and Gata3-tg mice 2 months after DA virus infection. Arrowheads show hippocampal atrophy of the region CA1. Tissue sections are representative of nine wild-type mice and seven Gata3-tg mice and representative of two independent experiments. Scale bar = 300 μm (brain sections) and 200 μm (spinal cord sections).
